# Association of Exposure to Phthalates with Endometriosis and Uterine Leiomyomata: Findings from NHANES, 1999–2004

**DOI:** 10.1289/ehp.0901543

**Published:** 2010-02-25

**Authors:** Jennifer Weuve, Russ Hauser, Antonia M. Calafat, Stacey A. Missmer, Lauren A. Wise

**Affiliations:** 1 Rush Institute for Healthy Aging, Rush University Medical Center, Chicago, Illinois, USA; 2 Department of Environmental Health, Harvard School of Public Health, Boston, Massachusetts, USA; 3 Vincent Memorial Obstetrics and Gynecology Service, Andrology Laboratory and *In Vitro* Fertilization Unit, Massachusetts General Hospital, Boston, Massachusetts, USA; 4 Centers for Disease Control and Prevention, Atlanta, Georgia, USA; 5 Department of Obstetrics, Gynecology, and Reproductive Medicine and; 6 Channing Laboratory, Department of Medicine, Brigham and Women’s Hospital, Harvard Medical School, Boston, Massachusetts, USA; 7 Department of Epidemiology, Harvard School of Public Health, Boston, Massachusetts, USA; 8 Slone Epidemiology Center, Boston University, Boston, Massachusetts, USA

**Keywords:** dibutyl phthalate, di(2-ethylhexyl) phthalate, endometriosis, leiomyomata, monobenzyl phthalate, monobutyl phthalate, monoethyl phthalate, mono(2-ethyl-5-hydroxyhexyl) phthalate, mono(2-ethyl-5-oxohexyl) phthalate, mono(2-ethylhexyl) phthalate

## Abstract

**Background:**

Phthalates are ubiquitous chemicals used in consumer products. Some phthalates are reproductive toxicants in experimental animals, but human data are limited.

**Objective:**

We conducted a cross-sectional study of urinary phthalate metabolite concentrations in relation to self-reported history of endometriosis and uterine leiomyomata among 1,227 women 20–54 years of age from three cycles of the National Health and Nutrition Examination Survey (NHANES), 1999–2004.

**Methods:**

We examined four phthalate metabolites: mono(2-ethylhexyl) phthalate (MEHP), monobutyl phthalate (MBP), monoethyl phthalate (MEP), and monobenzyl phthalate (MBzP). From the last two NHANES cycles, we also examined mono(2-ethyl-5-hydroxyhexyl) phthalate (MEHHP) and mono(2-ethyl-5-oxohexyl) phthalate (MEOHP). We used logistic regression to estimate odds ratios (ORs) and 95% confidence intervals (CIs), adjusting for potential confounders.

**Results:**

Eighty-seven (7%) and 151 (12%) women reported diagnoses of endometriosis and leiomyomata, respectively. The ORs comparing the highest versus lowest three quartiles of urinary MBP were 1.36 (95% CI, 0.77–2.41) for endometriosis, 1.56 (95% CI, 0.93–2.61) for leiomyomata, and 1.71 (95% CI, 1.07–2.75) for both conditions combined. The corresponding ORs for MEHP were 0.44 (95% CI, 0.19–1.02) for endometriosis, 0.63 (95% CI, 0.35–1.12) for leiomyomata, and 0.59 (95% CI, 0.37–0.95) for both conditions combined. Findings for MEHHP and MEOHP agreed with findings for MEHP with respect to endometriosis only. We observed null associations for MEP and MBzP. Associations were similar when we excluded women diagnosed > 7 years before their NHANES evaluation.

**Conclusion:**

The positive associations for MBP and inverse associations for MEHP in relation to endometriosis and leiomyomata warrant investigation in prospective studies.

Phthalates are a class of organic chemicals that have been used as plasticizers for polyvinylchloride formulations since the 1930s. These chemicals have been widely used in food packaging, medical devices and medications, toys, and building materials, as well as cosmetics and personal care products commonly used by women, such as perfume, lotion, and nail polish (Blount et al. 2000b). There is increasing concern that phthalates may have negative effects on human fertility and reproductive health, although there have been few studies on this topic. Moreover, most of the human studies suggesting an effect of phthalates on reproductive health have focused on males and have found deleterious effects on testicular function and spermatogenesis (Hauser and Calafat 2005).

Several studies in experimental animals suggest that phthalates possess endocrine-disrupting properties and can adversely affect reproductive function by antagonizing or altering the effects of endogenous sex steroid hormones (Agarwal et al. 1985, 1989; Arcadi et al. 1998; Berman and Laskey 1993; Davis et al. 1994a, 1994b; Lamb et al. 1987; Laskey and Berman 1993; Lovekamp and Davis 2001), and an emerging literature has begun to identify potential effects on female animals. Specifically, studies of sexually mature rats have found that exposure to di(2-ethylhexyl) phthalate (DEHP) and one of its metabolites, mono(2-ethylhexyl) phthalate (MEHP), is associated with smaller preovulatory follicles, anovulation or delayed ovulation, longer estrous cycles, decreased synthesis of estradiol, decreased serum progesterone levels, and increased serum follicle-stimulating hormone (FSH) levels (Davis et al. 1994a, 1994b; Lovekamp and Davis 2001). In addition, studies in pregnant and pseudopregnant rats have found associations between exposures to dibutyl phthalate (DBP) and benzyl butyl phthalate (BzBP) and impaired implantation in mated females (Ema et al. 1998, 2000), suggesting a possible link between phthalates and alterations in the endogenous hormonal milieu. In these studies, monobutyl phthalate (MBP), the main metabolite of DBP, and other phthalate metabolites, including monomethyl phthalate, monoethyl phthalate (MEP), monopropyl phthalate, and monopentyl phthalate, had no effect on estradiol synthesis (Lovekamp and Davis 2001).

Given that some phthalates have antiestrogenic activities (Davis et al. 1994a, 1994b; Lamb et al. 1987), and some have been shown to have weak estrogenic activity *in vitro* (Harris et al. 1997; Picard et al. 2001), there is biologic plausibility for an effect of phthalates on the incidence of hormone-responsive conditions such as endometriosis and uterine leiomyomata (fibroids). Endometriosis and uterine leiomyomata are common disorders among reproductive-age women and are associated with considerable morbidity, including pelvic pain, menorrhagia, and infertility (Eskenazi and Warner 1997; Schwartz et al. 2000; Velebil et al. 1995). Endometriosis is characterized by growth beyond or outside the uterus of tissue resembling the endometrium. Uterine leiomyomata are benign neoplasms composed of smooth muscle cells, growing within or around the myometrium. Prevalence estimates of clinically relevant endometriosis and leiomyomata in premenopausal women are approximately 10% and 30%, respectively (Baird et al. 2003; Missmer and Mohllajee 2008; Schwartz et al. 2000). Both conditions are leading indications for hysterectomy in the United States (Farquhar and Steiner 2002; Wilcox et al. 1994) and are associated with significant costs for lost productivity and hospitalization, accounting for several billion dollars annually in the United States for health care alone (Flynn et al. 2006; Simoens et al. 2007). Although the etiologies of both conditions remain poorly understood, there is evidence that they are influenced by endogenous sex steroid hormones (Eskenazi and Warner 1997; Schwartz et al. 2000).

Four epidemiologic studies have assessed phthalate exposures in relation to these conditions, and their findings suggest increased risk with higher levels of exposure to some phthalates (Cobellis et al. 2003; Itoh et al. 2009; Luisi et al. 2006; Reddy et al. 2006). However, these studies were small (with 15–57 cases), adjusted for few if any potential confounders, and recruited controls from clinical settings, a potential source of bias. Moreover, most of these studies relied on plasma or serum levels of phthalate diesters and monoesters as biomarkers of exposure. These exposure measures have two critical limitations. First, measurements of diesters in any biologic medium are prone to contamination from phthalate-containing laboratory supplies and other sources. Second, monoester measurements in blood are also susceptible to contamination, because serum enzymes can hydrolyze diesters in the blood to their respective monoesters after the specimen has been collected (Kato et al. 2003). The potential for contamination generally decreases when measuring phthalate monoester metabolites in urine. In assessing exposure to DEHP, the potential for contamination is virtually eliminated when measuring the metabolites produced via enzymatic oxidation (Kato et al. 2004).

Therefore, we examined the association of phthalate exposures with endometriosis and uterine leiomyomata, using cross-sectional data on urinary phthalate metabolite concentrations and self-reported reproductive history from 1,227 women participating in the National Health and Nutrition Examination Survey (NHANES), 1999–2004. In addition, for 838 women, we examined urinary concentrations of two oxidative metabolites of DEHP, which were measured in NHANES 2001–2004.

## Materials and Methods

We used data from three cycles (1999–2000–2001–2002–2003–2004) of NHANES [Centers for Disease Control and Prevention (CDC) 2009] to examine the association between urinary phthalate metabolites and previously diagnosed endometriosis or uterine leiomyomata. Briefly, NHANES is an ongoing cross-sectional national survey conducted by the National Center for Health Statistics at the CDC, designed to collect data on the health and nutritional status of the civilian, noninstitutionalized U.S. population. Data estimates are sampling-probability based and are representative of the U.S. population. NHANES collects data through questionnaires, physical examinations, and laboratory tests. The assessment includes both a household interview and mobile examination component (MEC), during which blood and urine are collected. Sociodemographic information and medical histories of the survey participants and their families were collected during the household interviews (CDC 2009). All participants provided written informed consent, and the NCHS obtained institutional review board approval to conduct the survey.

### Study population

NHANES 1999–2004 was conducted in 87 locations throughout the United States and included examinations of 31,126 people of all ages (15,942 females). A random one-third subset of participants was selected by NHANES for phthalate metabolite measurements, thereby retaining the representational aspect of these data (CDC 2009).

Women participating in the MEC assessment and who completed the reproductive health questionnaire (given to all female participants ≥ 12 years of age) were eligible for our analyses (*n* = 10,946). Questions pertaining to endometriosis and leiomyomata were restricted to women 20–54 years of age, further restricting the eligible population to 4,094 women. Of these women, 1,379 were included in the one-third subset of participants whose urinary specimens were analyzed for phthalates. We further excluded the 127 women whose urinary creatinine concentrations were < 30 or > 300 mg/dL (Silva et al. 2004). An additional 25 women were missing data on key covariates, leaving 1,227 women for our analyses of endometriosis. Of these women, six were missing responses pertaining to uterine leiomyomata, and thus our analyses of this outcome included 1,221 women. Because menopausal status is unlikely to influence the measurement of urinary phthalate metabolites, we retained women who were postmenopausal at the time of the MEC interview. Our analyses of two oxidative metabolites of DEHP (assessed in NHANES 2001–2004) in association with endometriosis and uterine leiomyomata, included 838 and 835 women, respectively.

Women who were excluded from our main analyses did not differ substantially from included women with respect to a range of demographic, lifestyle, and reproductive health factors. For example, these groups were similar in age (35.6 vs. 35.7 years), age at menarche (12.6 vs. 12.6 years), parity status (25% vs. 27% nulliparous), and race/ethnicity (19% vs. 21% African American).

### Assessment of exposure to phthalates

We examined the metabolites of four prevalent phthalates measured in the three consecutive NHANES cycles: MEHP, a monoester metabolite of DEHP; MBP; MEP, metabolite of diethyl phthalate (DEP); and monobenzyl phthalate (MBzP), the main metabolite of BzBP. In the 2001–2002 and 2003–2004 cycles, concentrations of mono-*n*-butyl phthalate (MnBP) and mono-isobutyl phthalate were measured separately, and we summed these concentrations to obtain total MBP concentration for these cycles. Data for other phthalate metabolites—monocyclohexyl phthalate, mono-*n*-octyl phthalate, and monoisononyl phthalate—were also available for the three NHANES cycles. However, these metabolites were detected in < 10% of the samples examined and were not included in our analyses.

Urinary concentrations of additional phthalate metabolites were introduced in the 2001–2002 cycle. Of particular interest to our study were concentrations of two oxidative metabolites of DEHP: mono(2-ethyl-5-hydroxyhexyl)phthalate (MEHHP) and mono(2-ethyl-5-oxohexyl) phthalate (MEOHP). These metabolites are produced only via enzymatic oxidation and are not produced in the environment. Moreover, their concentrations in urine are often at least an order of magnitude greater than concentrations of MEHP, making them potentially more sensitive measures of exposure to DEHP. Therefore, we also examined these two oxidative metabolites for data in the 2001–2002 and 2003–2004 cycles to evaluate the consistency of the findings for MEHP. As a summary measure of DEHP exposure, we computed the molar sum concentration of MEHP, MEHHP, and MEOHP. In exploratory analyses, we evaluated a fourth metabolite of DEHP, the oxidative metabolite, mono(2-ethyl-5-carboxypentyl) phthalate (MECPP), which was measured only in the 2003–2004 cycle.

Urine specimens for analyses, including phthalate metabolites and creatinine concentrations, were collected from each participant during one of three daily examination periods (0830–1200, 1230–1600, or 1630–2000 hours) (Silva et al. 2004). After collection, urine specimens were aliquoted and stored cold (2–4°C) or frozen until they were shipped to the various laboratories for analysis (Silva et al. 2004). Urine specimens were analyzed for creatinine using a Beckman Synchron AS/ASTRA clinical analyzer (Beckman Instruments, Inc., Brea, CA) at the University of Minnesota Medical Center (Silva et al. 2004). Samples collected for phthalate metabolite measurements were shipped on dry ice to the CDC’s National Center for Environmental Health. Urine samples were stored frozen at or below −20°C until analyzed. The samples were analyzed by solid-phase extraction followed by isotope-dilution high-performance liquid chromatography and tandem mass spectrometry as previously described (Blount et al. 2000a; Silva et al. 2003). To correct for urine dilution, we divided all phthalate metabolite concentrations by urinary creatinine concentration (Jackson 1966) and used the resulting creatinine-corrected concentrations for our primary analyses. Concentrations below the limit of detection (LOD) were replaced with the LOD divided by the square root of 2 (Hornung and Reed 1990). For any given urinary metabolite, the proportion of women in our study who had concentrations < LOD ranged from 0.2% to 20.5%.

### Assessment of endometriosis and uterine leiomyomata

We classified each woman’s history of endometriosis and uterine leiomyomata according to her response to two questions: *a*) “Has a doctor or other health professional ever told you that you had endometriosis?” *b*) “Has a doctor or other health professional ever told you that you had uterine fibroids?” Women who answered positively to either of these questions were further queried regarding their age at first diagnosis (open-ended question) (CDC 2009).

### Assessment of covariates

Data on age at menarche, gravidity, parity, current pregnancy status, breast-feeding, use of hormonal contraception, menopausal status, and gynecologic surgeries were obtained from the NHANES reproductive health questionnaire. We obtained data on age and race/ethnicity from the NHANES home interview.

### Statistical analysis

We conducted all statistical analyses using SAS-callable SUDAAN, release 9.0.2 (RTI International, Research Triangle Park, NC). Unless specified, our analyses accounted for the complex sampling design of and nonresponse to the NHANES through use of sampling weights and sample design variables. Sampling errors were estimated using the Taylor series linearized method (CDC 2005).

We computed geometric mean phthalate metabolite concentrations within diagnosis groups, testing differences by case status using linear regression models of log-transformed phthalate concentrations. Within quartiles of each phthalate metabolite and within case status for each of the two outcomes, we determined means and frequencies of various characteristics, testing differences using linear and logistic regression models. To evaluate the association between exposure to phthalates and the two outcomes, we used multiple logistic regression to derive adjusted odds ratios (ORs) and their 95% confidence intervals (CIs) across quartiles of each phthalate metabolite concentration in relation to endometriosis and uterine leiomyomata. Tests for trend were calculated by including in the regression model a single variable coded as the median of each exposure quartile (Breslow and Day 1987). We conducted separate analyses for each phthalate metabolite and for each outcome, comparing women who reported a history of the outcome with those who did not. Because endometriosis and uterine leiomyomata share some common hormonal characteristics, we also analyzed a combined end point defined as having a history of one or both conditions, allowing for increased statistical power, the caveat being that associations could be diluted if the phthalate-related etiologies differ by outcome. Because the distributions of the urinary phthalate concentrations were strongly right-skewed, we also conducted analyses in which we compared women in the highest quartile of each metabolite with those in the lowest three quartiles.

As potential confounders, we examined variables that were associated with urinary phthalate concentrations in our study population and are also considered well-established risk factors for the outcomes of interest. Final models were adjusted for age (years) at time of NHANES urine sample collection, race/ethnicity (African American, white, other), age at menarche (years), and both current pregnancy and breast-feeding status. Because of the possibility that hysterectomy, nulliparity, and oral contraceptive use could be consequences of the outcomes, we did not adjust for these variables. For models involving only 2001–2004 data, we did not adjust for breast-feeding status, because very few women in this population (*n* = 28) reported breast-feeding. We were unable to consider infertility history, because these data were not publicly available.

The exposure interval directly reflected by urinary phthalate concentrations is quite short. To evaluate the associations between phthalate exposures and the two outcomes in a manner that better reflected the exposures that were actually measured, we conducted secondary analyses that excluded women who had the diagnosis of interest or a hysterectomy > 10 years before their NHANES evaluation [leaving *n* = 1,146 (47 cases) and 1,134 (96 cases) for analyses of endometriosis and uterine leiomyomata, respectively] and > 7 years before [leaving *n* = 1,128 (38 cases) and 1,112 (79 cases) for endometriosis and leiomyomata, respectively]. For the analyses of uterine leiomyomata and the combined end point, we had sufficient cases to additionally make these exclusions on the basis of a 5-year interval [leaving *n* = 1,096 (67 cases) for leiomyomata analyses].

We performed several sensitivity analyses. In parallel to our primary analyses of creatinine-corrected urinary phthalate metabolite concentrations, we conducted analyses using raw phthalate metabolite concentrations as the measures of exposure and then included urinary creatinine as an additional term in the logistic regression models. We also reanalyzed the data, allowing for the inclusion of the 127 women with very low (< 30 mg/dL) or very high (> 300 mg/dL) urinary creatinine concentrations. Finally, although our analyses of MEHHP and MEOHP constitute a sensitivity analysis of the MEHP analyses, for enhanced comparability, we evaluated MEHP over the same period in which the oxidative metabolites were assessed (2001–2004).

We considered a *p*-value < 0.05 to be statistically significant. For all analyses, we used creatinine-corrected urinary concentrations of the phthalate metabolites, unless stated otherwise.

## Results

Among the 1,227 women included in our analysis, the unweighted percentages of those 20–29, 30–39, 40–49, and 50–54 years of age were, respectively, 33.8%, 27.1%, 27.8% and 11.3%. Eighty-seven women (7%) reported a previous diagnosis of endometriosis, 151 (12%) reported a previous diagnosis of uterine leiomyomata, and 201 (16%) reported a previous diagnosis of either condition [34 (3%) had both conditions]. Women with a history of endometriosis reported that their diagnoses occurred < 1 year to 34 years before their NHANES evaluation (median, 9 years). For uterine leiomyomata, this range was < 1 to 36 years (median, 7 years).

Table 1 lists the geometric mean urinary concentrations of the phthalate metabolites. Urinary concentrations of MBP were somewhat higher among women with endometriosis, and women with endometriosis or leiomyomata had significantly lower concentrations of MEHP than did women without these conditions (*p* = 0.03).

Women in the highest quartiles of urinary MEP were significantly more likely to be non-Hispanic black or Mexican American than were women in the lowest quartiles (Table 2). Women in the lowest MEP quartile were more likely to be currently breast-feeding than were women in the higher quartiles. Women in the highest quartile of urinary MBzP were significantly younger, less likely to be Mexican-American, and more likely to be pregnant than were women in the lower quartiles. None of the characteristics varied significantly across quartiles of urinary MEHP, but some patterns emerged in data on the DEHP oxidative metabolites (data not shown). For example, compared with the lowest quartiles of urinary MEOHP, women in the highest quartile were more likely to be non-Hispanic white (*p* = 0.02).

Women who reported a history of endometriosis were significantly older, more likely to be non-Hispanic white, and less likely to be non-Hispanic black or Mexican American than were noncases (Table 2). They were also significantly less likely to have been currently breast-feeding. Women who reported a history of uterine leiomyomata were also significantly older than noncases, but in contrast to the race/ethnicity pattern for endometriosis, leiomyomata cases were significantly more likely than noncases to be non-Hispanic black. Nulliparity and current use of oral contraceptives were significantly less common among leiomyomata cases than among noncases. History of hysterectomy was common among women who reported either condition.

Urinary concentration of MBP was weakly associated with increased odds of both endometriosis (highest vs. lowest three quartiles: OR = 1.36; 95% CI, 0.77–2.41) and leiomyomata (highest vs. lowest three quartiles: OR = 1.56; 95% CI, 0.93–2.61) (Table 3). When we examined the outcomes as a combined end point, the OR comparing the highest versus lowest three quartiles of urinary MBP was 1.71 (95% CI, 1.07–2.75). Urinary concentration of MEHP was inversely associated with both endometriosis (highest vs. lowest three quartiles: OR = 0.44; 95% CI, 0.19–1.02) and leiomyomata (highest vs. lowest three quartiles: OR = 0.63; 95% CI, 0.35–1.12). For endometriosis, ORs decreased significantly with progressively higher levels of MEHP (*p*_trend_ = 0.05). For the outcomes combined, we found statistically significant inverse associations corresponding to both the contrast between highest and lowest three quartiles of MEHP (OR = 0.59; 95% CI, 0.37–0.95) and the trend across increasing quartiles of MEHP (*p*_trend_ = 0.03).

The associations of urinary MBP and MEHP concentrations with these outcomes either remained unchanged or became stronger when we excluded women whose diagnoses or hysterectomy occurred further in the past. For MBP and leiomyomata, when we excluded women whose leiomyomata diagnosis or hysterectomy occurred > 7 years before their NHANES examination, the OR for women in the highest quartile of MBP concentration versus those in the lowest three quartiles increased to 1.86 (95% CI, 0.94–3.68). The corresponding OR for the combined outcome increased to 2.16 (95% CI, 1.23–3.77), with similar findings with exclusions based on a 5-year interval from diagnosis. For MEHP and endometriosis, when we excluded women whose diagnosis or hysterectomy occurred > 7 years before their NHANES examination, the OR for women in the highest quartile of MEHP concentration versus those in the lowest three quartiles decreased to 0.19 (95% CI, 0.05–0.78). For analyses of MEHP and the combined outcome, when we made exclusions based on a 5-year interval, the findings were consistent with those from our main analyses.

In analyses of the oxidative metabolites of DEHP (MEHHP and MEOHP), we found associations that were consistent with that between MEHP and endometriosis, but not with that between MEHP and leiomyomata (Table 4). Specifically, the OR of endometriosis for women in the highest quartile of MEHHP concentration versus those in the lowest three quartiles was 0.46 (95% CI, 0.18–1.21), and the comparable ORs for MEOHP and the molar sum of MEHP, MEHHP, and MEOHP also indicated an inverse association. In contrast, concentrations of the oxidative metabolites and the molar sum were not consistently associated with leiomyomata. Results for the highest versus the lowest three quartiles of MECPP, measured only in 2003–2004, were largely consistent with the pattern of these findings, with OR = 0.49 (95% CI, 0.12–1.93) for endometriosis (57 cases) and OR = 1.63 (95% CI, 0.89–2.96) for uterine leiomyomata (113 cases).

Urinary concentrations of MEP and MBzP were not appreciably associated with risk of endometriosis or leiomyomata. Because current pregnancy was more common with higher urinary MBzP and less common in both outcomes, we repeated analyses of MBzP excluding pregnant women, but the results were similar.

Moreover, our results overall did not change after further adjustment for parity or use of oral contraceptives or when we excluded women who used oral contraceptives. They also did not change substantially when we replaced the creatinine-corrected urinary phthalate concentrations with uncorrected concentrations or when we included women with extreme urinary creatinine concentrations.

## Discussion

In this large nationally representative cross-sectional study, in which we used urinary phthalate metabolite concentrations as biomarkers of exposure to four parent phthalates, we observed weak positive associations for urinary MBP, and weak inverse associations for urinary MEHP, in relation to self-reported history of endometriosis and leiomyomata. Our analyses of the two oxidative metabolites of DEHP—MEHHP and MEOHP—yielded findings that were consistent with our findings for MEHP with respect to endometriosis, but not with respect to uterine leiomyomata. We found little evidence for associations of urinary MEP and MBzP with each of these outcomes.

Our results for MEHP and endometriosis do not agree with findings from the three case–control studies on this subject, all which identified positive associations between markers of DEHP exposure and risk of endometriosis. Cobellis et al. (2003) reported higher plasma DEHP concentrations [median, 0.57 μg/mL; interquartile range (IQR), 0.06–1.23] among women with endometriosis than among controls (0.18 μg/mL; IQR, 0–0.44; *p* = 0.005). However, plasma MEHP concentrations were generally low and did not differ between the outcome groups: for cases the median MEHP was 0.38 μg/mL (IQR, 0.1–0.97), and for controls the median was 0.58 μg/mL (IQR, 0.34–0.71). Our MEHP results also diverge from a subsequent case–control study by Reddy et al. (2006), in which women with laparoscopy-confirmed endometriosis had significantly higher plasma concentrations of DEHP compared with controls, and concentrations appeared more elevated with increasingly severe stage of endometriosis. In a third case–control study by Itoh et al. (2009), laparoscopy-confirmed cases were more likely than controls to have “elevated” creatinine-corrected urinary MEHP concentrations [above the controls’ median (4.2 ng/mg)], although this association was not significant. Findings were weaker for urinary MEHHP, MEOHP, and the molar sum of the three DEHP metabolites. Itoh et al. also found significantly higher uncorrected concentrations of all three DEHP metabolites with increasingly severe stage of endometriosis (*p* < 0.02), but these trends diminished upon creatinine correction. The discrepant findings may stem from differences in exposure assessment and/or case definition. For measures of exposure to DEHP, the first two case–control studies used blood-based concentrations of DEHP, which is no longer considered a valid approach for assessing exposure because of concerns about contamination from laboratory equipment and other external sources. The correlation between serum DEHP and MEHP concentrations was weak in the Cobellis et al. (2003) study (*r* = 0.16, *p* ≥ 0.3), raising questions about the utility of the serum DEHP measurements, including the possibility that DEHP measurements were affected by external contamination (Hauser and Calafat 2005). Blood-based concentrations of MEHP are also prone to contamination from DEHP that is also present in the specimen, because serum enzymes can hydrolyze DEHP to MEHP during storage (Kato et al. 2003). For their case definitions, all three case–control studies enrolled women with laparoscopically confirmed endometriosis. Cases in the Cobellis et al. (2003) study underwent further histologic confirmation. However, because all participants were enrolled in clinical settings, it is possible that DEHP and MEHP concentrations were affected by supplies used in very recent medical procedures associated with the clinical conditions (Hauser and Calafat 2005). For example, at least some of the specimens in the Cobellis et al. (2003) study were collected immediately before anesthesia for laparoscopy, when the women were likely on an intravenous therapy line, a potential source of DEHP exposure (U.S. Food and Drug Administration 2002). This may explain why plasma MEHP concentrations in the Cobellis et al. (2003) study were about 100 times the urinary MEHP concentrations among the women in our study. It is also possible that controls recruited from a clinical setting—such as those seeking treatment for infertility in the Itoh et al. (2009) study—had conditions that were themselves influenced by exposure to phthalates, leading to biased findings (Hernán et al. 2004).

In contrast, our results for MBP agree with results from the Reddy et al. (2006) study in which women with endometriosis had significantly higher plasma concentrations of DBP (the parent compound of MBP) compared with controls (Reddy et al. 2006). Nonetheless, blood measures of DBP entail limitations as described for DEHP, and even in the absence of external contamination, there remains the possibility that some of the DBP could be missed because of its hydrolysis to MBP (Kato et al. 2003). In the study by Itoh et al. (2009), endometriosis was not significantly associated with having an “elevated” creatinine-corrected urinary MnBP concentration (above the median among the controls, 43.4 ng/mg), and there was no apparent trend in urinary MnBP across severity classes of the condition.

Our null findings for MBzP and endometriosis were consistent with those of Itoh et al. (2009) but diverged from those of Reddy et al. (2006) in which plasma BzBP concentrations among cases were significantly higher than those among controls. Similar to our findings for MEP, Itoh et al. (2009) did not find a significant association between creatinine-corrected urinary MEP and endometriosis.

Our weakly inverse association between MEHP and leiomyomata agrees with the case–control study by Luisi et al. (2006), in which significantly lower serum DEHP and MEHP concentrations were found among hysterectomy-confirmed cases of leiomyomata relative to controls (confirmed by ultrasound to be free of disease). The measurement concerns we described above also pertain to this case–control study, but they would likely serve to overestimate DEHP exposures among the cases, rendering the estimates from this case–control study conservative. However, this study may have reduced external contamination through its use of glass collection and storage materials.

Our results are also consistent with animal data showing positive associations of exposure to DEHP and its metabolite MEHP with anovulation or delayed ovulation, longer estrous cycles, decreased synthesis of estradiol, and decreased serum progesterone levels (Davis et al. 1994a, 1994b; Lovekamp and Davis 2001), all of which would predict a protective effect on uterine leiomyomata and endometriosis. The pathophysiology of leiomyomata is believed to depend on the biological activity of the endogenous sex steroid hormones estrogen (estradiol, estrone, estriol) and progesterone (Rein 2000; Rein and Nowak 1992; Rein et al. 1995), as well as locally derived growth factors (Andersen 1998). The hormone-dependent nature of leiomyomata is supported by the following observations: They do not occur before menarche, they have an increased concentration of estrogen and progesterone receptors compared with normal myometrium (Strauss and Coutifaris 1999), and they shrink in volume after menopause or with suppression of ovarian function via gonadotropin-releasing hormone (GnRH) agonist therapy or oophorectomy (Friedman et al. 1990). The literature on the relation of endogenous sex hormones to leiomyomata is limited and mixed. Although an earlier study reported than serum estrogen levels did not differ significantly between leiomyomata cases and controls (Potgieter et al. 1995), a subsequent study reported that urinary concentrations of many sex hormones, including 17β-estradiol, were significantly higher among leiomyomata cases than controls (Jung et al. 2004). Several epidemiologic studies show an increased risk of leiomyomata associated with reproductive and hormonal factors, including early age at menarche and nulliparity, which suggests that increased menstrual cycling is a risk factor (Schwartz 2001; Wise et al. 2004).

Similarly, endometriosis is associated with abnormal steroid production and activity (Dizerega et al. 1980; Eskenazi and Warner 1997), with diagnoses occurring postmenarchally (Houston 1984) and symptoms abating after menopause. Endometriotic implants are dependent on estrogen for their maintenance and growth (Dizerega et al. 1980; Gurates and Bulun 2003), and reduction of estradiol production, via either surgical (oophorectomy) or hormonal (GnRH agonist analogues) intervention, causes atrophy of endometriosis lesions and is effective in treating pain symptoms. High-dose synthetic progestins are effective in the treatment of endometriosis through suppression of luteinizing hormone and FSH secretion that inhibits estradiol production, direct antiestrogenic effects on the lesions, and induction of pseudodecidualization (Taylor and Lebovic 2009). Estradiol production is up-regulated in endometriotic implants, whereas estradiol metabolism is impaired because of a deficiency of the enzyme 17β-hydroxysteroid dehydrogenase type 2 (Gurates and Bulun 2003). Also, the progesterone receptor subtype B is deficient in endometriosis tissue (Attia et al. 2000). In epidemiologic studies, endometriosis is positively associated with early age at menarche, shorter cycle length, decreased parity, and not breast-feeding (Missmer et al. 2004a), all of which can affect menstrual cycling and lifetime cumulative exposure to estrogens.

Nonetheless, although our findings for DEHP’s oxidative metabolites provided support for an inverse association between DEHP exposure and endometriosis, they raised doubts about an association with leiomyomata. In the subset of women evaluated in 2001–2004, we compared findings for urinary concentrations of MEHHP and MEOHP with findings for MEHP, and both MEHHP and MEOHP were detectable in nearly all specimens in our study, including those in which MEHP was undetectable. The associations of the two oxidative metabolite concentrations with endometriosis were inverse and remarkably similar in magnitude to the corresponding association for MEHP. However, the associations between the oxidative metabolites and uterine leiomyomata were not consistent with the inverse (albeit nonsignificant) corresponding association for MEHP. The association for MEHHP was essentially null [OR = 0.97 (95% CI, 0.55–0.71) for women in the highest quartile vs. the lowest three quartiles], and although the associations for MEOHP and MECPP suggested a positive (nonsignificant) association, further analyses at alternative threshold concentrations were more consistent with null associations [e.g., OR = 0.92 (95% CI, 0.39–2.15) comparing highest sextile of urinary MEOHP vs. the lowest five]. Although these findings emanated from a subset of participants, they suggest that the inverse association between MEHP and leiomyomata may be a chance finding.

Our study has three principal limitations that warrant consideration. First, with its cross-sectional design, the assessment of exposure to phthalates may have occurred several years after diagnosis of the outcomes. It is uncertain whether diagnosis would directly influence urinary phthalate concentrations by altering the metabolism of phthalates, although recent data from 60 women in the Early Pregnancy Study indicate little variation in urinary phthalate metabolite concentrations across phases of the menstrual cycle (Baird et al. 2009), suggesting that changes in a woman’s endogenous hormonal milieu (which might occur with age, oral contraceptive use, or hysterectomy) have minimal effects on phthalate metabolism. However, it remains possible that diagnoses could indirectly affect urinary phthalate concentrations via exposures resulting from diagnosis, as described above. However, given the short half-lives of the metabolites, it seems unlikely that diagnosis-related procedures occurring more than a few days before the NHANES examination would be an important source of exposure. Moreover, although medications have recently been identified as an important source of DBP and DEP exposures (Hauser et al. 2004; Hernández-Díaz et al. 2009), medications typically indicated for endometriosis and leiomyomata are not likely to contain these compounds because they are not coated for timed release. Even in the absence of reverse causation, the increasing interval between the relevant and measured exposures is a form of measurement error—nondifferential with respect to exposure or disease status—that could attenuate the observed associations. The associations between MBP and uterine leiomyomata and between MEHP and endometriosis became stronger when we confined analyses to women whose diagnoses or hysterectomies occurred within 7 years of their NHANES evaluation, suggesting that this type of exposure measurement error may have affected our results.

Our exposure measure is limited in that phthalates do not bioaccumulate and have short half-lives in the body. Therefore, measurements taken at a single point in time are less reliable indicators of typical exposures than, for example, a series of measurements taken over several weeks. Yet emerging data suggest some consistency of phthalate exposures over time among reproductive-age women. In their study of 46 women 35–49 years of age who provided first-morning urinary voids on 2 consecutive days, Hoppin et al. (2002) found intraclass correlations (ICCs) ranging from 0.53 to 0.80 for creatinine-corrected urinary concentrations of MBP, MEP, MEHP, and MBzP. Over longer time intervals, these correlations may be weaker, as suggested by data from two studies: the Early Pregnancy Study, which reported ICCs for these urinary metabolites ranging from 0.36 to 0.53 for measurements taken over 2-week intervals (Baird et al. 2009); and a study of 28 pregnant women (Adibi et al. 2008), which reported ICCs for the creatinine-corrected urinary phthalate concentrations ranging from 0.21 to 0.65 for measurements taken repeatedly over 6 weeks. Therefore, it is possible that we have underestimated the true associations, where they exist, between phthalate exposures and endometriosis and uterine leiomyomata. Nonetheless, our exposure measures do represent an advance over measures used in previous work in that we have used urinary measures of phthalate metabolites, which are far less vulnerable to contamination than phthalate diester measurements in blood. Moreover, we have evaluated exposures to four phthalates, conferring a degree of specificity to our findings.

Finally, an important limitation of our study is that measures of both outcomes were based solely on self-report of physician diagnosis. Prevalence estimates of these conditions in our study population are similar to studies based on hospital-discharge and prospective cohort data (Eskenazi and Warner 1997; Missmer and Cramer 2003; Schwartz and Marshall 2000; Wise et al. 2005). Two large prospective studies of self-reported leiomyomata that validated the reports against medical records indicated a low proportion of false positives (Marshall et al. 1997; Wise et al. 2004); therefore, specificity of diagnosis is likely to be high. False reports of endometriosis diagnosis may occur more frequently (Missmer et al. 2004b), but the proportion of undiagnosed endometriosis cases is likely to be smaller (Zondervan et al. 2002). For both outcomes, it seems unlikely that misclassification of disease status was influenced by degree of phthalate exposure, and such nondifferential misclassification would have attenuated our findings. Our findings for endometriosis may have been further influenced by phthalate effects that are specific to different types of underlying pathology. Estrogen-related risk factors appear to vary by endometriosis that is concurrent with and independent of infertility (the latter which is often accompanied by pain) (Missmer et al. 2004a, 2004b). However, in the absence of data on infertility and symptomatic pain, the findings reflect an averaged association of the phthalate measures with these two subgroups.

In addition to our study’s strengths in its exposure measurements, our study also benefited from its large sample size, its consideration of several important potential confounders, and its representativeness of the general population of reproductive-age women living in the United States. In conclusion, we have identified preliminary evidence suggesting that exposure to DBP may be associated with increased risk of endometriosis and uterine leiomyomata, and that exposure to DEHP may be associated with reduced risk of these conditions. These findings warrant investigation in future prospective studies.

## Figures and Tables

**Table 1:** Geometric mean urinary creatinine and creatinine-corrected urinary concentration of phthalate metabolites, by diagnosis.^a^

	Geometric mean (SE)				
	All women (*n*= 1,227)	Women with endometriosis (*n*= 87)	Women with uterine leiomyomata (*n*= 151)	Women with neither condition (*n*= 1,020)	Percentage of all women with levels < LOD^b^
Urinary creatinine (mg/dL)	108.2 (2.5)	108.4 (6.3)	114.0 (5.6)	107.3 (3.0)	0.0
Phthalate metabolites (ng/mg)					
MBP	26.2 (0.9)	28.9 (4.1)	27.1 (2.6)	25.5 (1.0)	0.5
MEP	216.2 (11.9)	207.0 (27.5)	210.2 (21.9)	219.9 (14.1)	0.2
MEHP	3.3 (0.1)	2.5 (0.4)	2.8 (0.3)	3.4 (0.1)*	20.5
MBzP	14.1 (0.6)	14.4 (2.5)	13.6 (1.2)	14.1 (0.6)	0.6
Oxidative metabolites of DEHP (ng/mg) (2001–2004)^c^					
MEHHP	19.4 (1.2)	16.5 (2.8)	20.0 (2.0)	19.7 (1.4)	0.8
MEOHP	13.3 (0.8)	11.5 (1.9)	13.8 (1.4)	13.5 (1.0)	1.7

a Numbers of women with and without a diagnosis do not sum to the total (1,227) because 34 women have both diagnoses, and data on diagnosis of uterine leiomyomata are missing for three women.

b Percentages for MEHHP and MEOHP are based on the 2001–2004 sample population (*n* = 838).

c *n* = 838, 57, 113, and 689 for all women, women with endometriosis, women with uterine leiomyomata, and women with neither condition, respectively.

* Significantly higher (*p* = 0.03) than among women with endometriosis or uterine leiomyomata.

**Table 2:** Characteristics of the study population, by quartile of creatinine-corrected urinary phthalate concentration and by diagnosis status.

				Race/ethnicity (%)				Gynecologic and reproductive history (%)							
	Quartile	Range (ng/mg)	Mean age	Non- Hispanic black	Non- Hispanic white	Mexican American	Other/mixed	Nulliparous	Currently pregnant	Currently breast-feeding	Age at menarche (years)			Currently uses oral contraceptives	History of hysterectomy
											< 12	12–13	> 13		
MBP	Lowest	0.4–15.6	37.5	10.9	72.3	8.1	8.7	25.5	4.5	4.7	24.7	51.3	24.0	14.5	10.7
	Second	15.7–25.3	36.1	13.2	72.1	8.6	6.1	30.0	8.0	1.8	23.1	54.0	22.9	14.4	11.8
	Third	25.4–41.3	37.5	11.7	64.0	8.1	16.2	26.6	4.9	2.0	23.2	54.3	22.5	12.6	10.6
	Highest	41.4–6,429	37.8	13.9	63.2	7.6	15.3	29.1	6.2	2.3	22.2	57.1	20.8	10.9	13.7

MEP	Lowest	0.6–100	37.6	8.8	73.4	4.7	13.1	28.0	5.6	5.6	24.0	58.4	17.6	11.7	11.2
	Second	101–209	36.9	11.8	72.3	6.3	9.6	27.0	4.9	2.3	27.0	50.4	22.7	13.1	12.3
	Third	210–517	37.3	14.0	62.9	10.8	12.3	26.4	6.4	0.9	22.3	51.1	26.6	12.3	14.7
	Highest	518–29,986	37.2	15.8*	61.1*	11.2*	11.8	29.9	6.8	1.7*	19.1	57.0	23.9	15.4	8.4

MEHP	Lowest	0.2–1.4	38.3	9.7	69.2	9.0	12.1	29.8	5.1	2.9	26.1	54.3	19.6	9.7	11.0
	Second	1.5–3.2	37.3	13.2	63.6	7.8	15.3	25.0	7.4	4.0	25.2	47.8	26.9	11.5	10.5
	Third	3.3–6.3	36.8	11.5	67.9	9.2	11.4	26.3	3.9	0.9	20.3	58.9	20.8	19.2	14.0
	Highest	6.4–538	36.4	15.4	70.8	6.3	7.4	29.9	7.0	2.7	21.1	56.4	22.5	12.5	11.5

MBzP	Lowest	0.2–7.7	38.9	12.9	67.4	10.5	9.3	32.2	4.7	2.1	22.7	52.1	25.2	14.6	10.5
	Second	7.8–13.1	37.4	12.1	65.2	8.8	10.9	28.7	3.6	2.6	24.7	53.2	22.1	15.4	9.7
	Third	13.2–23.2	36.9	12.1	67.5	4.5	12.9	26.1	5.6	2.4	20.4	57.6	22.0	11.6	14.4
	Highest	23.3–650	35.9*	12.6	71.1	5.8*	10.5	24.5	9.5*	3.6	25.5	53.5	21.0	10.9	12.1

Endometriosis	Yes		40.4	5.3	83.7	3.0	8.0	24.8	2.9	0.1	26.1	55.8	18.1	10.4	49.3
	No		36.9*	13.2*	66.2*	8.6*	12.0	28.1	6.2	3.0*	23.0	54.0	23.0	13.4	7.8*

Uterine leiomyomata	Yes		44.3	18.9	67.1	3.4	10.5	16.6	2.8	1.6	27.1	52.8	20.0	6.5	42.3
	No		36.0*	11.4*	67.9	8.1*	11.8	29.8*	6.4	2.9	22.8	54.4	22.8	14.2*	6.4*

* Difference across the quartiles or by diagnosis, *p* < 0.05.

**Table 3:** Adjusted^a^ ORs of endometriosis or uterine leiomyomata by concentration of creatinine-corrected urinary phthalate metabolite, in quartiles (1999–2004).

	MBP			MEP			MEHP			MBzP		
	Cases/*n*		OR (95% CI)	Cases/*n*		OR (95% CI)	Cases/*n*		OR (95% CI)	Cases/*n*		OR (95% CI)
Endometriosis												
Lowest Q	22/303		1.00 Reference	28/304		1.00 Reference	28/309		1.00 Reference	23/306		1.00 Reference
Second Q	20/306		0.75 (0.38–1.47)	20/309		0.89 (0.44–1.82)	21/303		0.78 (0.38–1.58)	19/306		0.84 (0.37–1.89)
Third Q	22/310		0.96 (0.49–1.91)	18/304		1.13 (0.56–2.27)	23/309		0.89 (0.42–1.90)	23/309		1.17 (0.47–2.94)
Highest Q	23/308		1.24 (0.65–2.34)	21/310		1.12 (0.53–2.35)	15/306		0.39 (0.16–0.95)	22/306		1.17 (0.42–3.27)
	*p*_trend_ = 0.3			*p*_trend_ = 0.6			*p*_trend_ = 0.05			*p*_trend_ = 0.6		
	Highest Q	Lowest three Qs	OR (95% CI)	Highest Q	Lowest three Qs	OR (95% CI)	Highest Q	Lowest three Qs	OR (95% CI)	Highest Q	Lowest three Qs	OR (95% CI)
	
Highest vs. lowest three Qs	23/308	64/919	1.36 (0.77–2.41)	21/310	66/917	1.12 (0.58–2.17)	15/306	72/921	0.44 (0.19–1.02)	22/306	65/921	1.16 (0.58–2.33)
Excluding women who had diagnosis or hysterectomy												
> 10 years ago	11/289	36/857	1.47 (0.70–3.10)	10/290	37/856	0.87 (0.30–2.51)	5/284	42/862	0.21 (0.06–0.74)	13/292	34/854	1.55 (0.55–4.37)
> 7 years ago	9/284	29/844	1.45 (0.64–3.28)	7/285	31/843	0.80 (0.23–2.76)	4/282	34/846	0.19 (0.05–0.78)	8/286	30/842	0.94 (0.28–3.12)

Uterine leiomyomata												
Lowest Q	33/301		1.00 Reference	41/301		1.00 Reference	43/307		1.00 Reference	37/306		1.00 Reference
Second Q	29/305		0.66 (0.40–1.10)	31/309		0.72 (0.35–1.46)	37/301		1.03 (0.59–1.80)	36/302		1.11 (0.59–2.07)
Third Q	39/308		0.76 (0.46–1.28)	45/303		1.29 (0.74–2.25)	42/307		1.13 (0.65–1.95)	47/309		1.16 (0.64–2.13)
Highest Q	50/307		1.26 (0.70–2.27)	34/308		0.85 (0.47–1.54)	29/306		0.66 (0.35–1.26)	31/304		1.14 (0.54–2.39)
	*p*_trend_= 0.2			*p*_trend_= 0.9			*p*_trend_= 0.2			*p*_trend_= 0.8		
	Highest Q	Lowest three Qs	OR (95% CI)	Highest Q	Lowest three Qs	OR (95% CI)	Highest Q	Lowest three Qs	OR (95% CI)	Highest Q	Lowest three Qs	OR (95% CI)
	
Highest vs. lowest three Qs	50/307	101/914	1.56 (0.93–2.61)	34/308	117/913	0.86 (0.51–1.46)	29/306	122/915	0.63 (0.35–1.12)	31/304	120/917	1.04 (0.57–1.92)
Excluding women who had diagnosis or hysterectomy												
> 10 years ago	30/281	66/853	1.61 (0.86–3.01)	25/291	71/843	1.10 (0.60–2.01)	17/284	79/850	0.60 (0.30–1.23)	22/291	74/843	0.97 (0.45–2.08)
> 7 years ago	26/274	53/838	1.86 (0.94–3.68)	20/285	59/827	1.14 (0.58–2.26)	14/281	65/831	0.57 (0.26–1.29)	21/288	58/824	1.22 (0.55–2.74)
> 5 years ago	23/269	44/827	1.87 (0.96–3.65)	19/283	48/813	1.35 (0.68–2.68)	12/278	55/818	0.60 (0.27–1.36)	19/284	48/812	1.33 (0.59–3.00)

Endometriosis or uterine leiomyomata												
Lowest Q	42/301		1.00 Reference	59/301		1.00 Reference	60/307		1.00 Reference	48/306		1.00 Reference
Second Q	41/305		0.74 (0.46–1.20)	44/309		0.75 (0.45–1.24)	50/301		0.93 (0.56–1.54)	47/302		1.01 (0.55–1.83)
Third Q	53/308		0.97 (0.62–1.53)	55/303		1.15 (0.75–1.78)	52/307		0.94 (0.57–1.55)	61/309		1.25 (0.70–2.24)
Highest Q	65/307		1.55 (0.87–2.75)	43/308		0.80 (0.49–1.30)	39/306		0.57 (0.33–0.98)	45/304		1.27 (0.59–2.72)
	*p*_trend_= 0.03			*p*_trend_= 0.6			*p*_trend_= 0.03			*p*_trend_= 0.5		
	Highest Q	Lowest three Qs	OR (95% CI)	Highest Q	Lowest three Qs	OR (95% CI)	Highest Q	Lowest three Qs	OR (95% CI)	Highest Q	Lowest three Qs	OR (95% CI)
	
Highest vs. lowest three Qs	65/307	136/914	1.71 (1.07–2.75)	43/308	158/913	0.83 (0.51–1.37)	39/306	162/915	0.59 (0.37–0.95)	45/304	156/917	1.16 (0.66–2.05)
Excluding women who had diagnosis or hysterectomy												
> 10 years ago	38/275	80/835	2.04 (1.12–3.45)	27/286	91/824	0.88 (0.50–1.54)	20/278	98/832	0.54 (0.28–1.02)	29/284	89/826	1.25 (0.62–2.52)
> 7 years ago	32/268	66/819	2.16 (1.23–3.77)	21/279	77/808	0.91 (0.48–1.72)	16/274	82/823	0.50 (0.24–1.02)	24/278	74/809	1.19 (0.60–2.34)
> 5 years ago	27/262	54/806	2.08 (1.14–3.80)	18/275	63/793	0.95 (0.50–1.80)	14/272	67/796	0.53 (0.25–1.13)	22/275	59/793	1.38 (0.66–2.87)

Q, quartile.

a Adjusted for age, race/ethnicity, age at menarche, current pregnancy status, and current breast-feeding status.

**Table 4:** Adjusted^a^ ORs of endometriosis or uterine leiomyomata by concentration of creatinine-corrected urinary phthalate metabolites of DEHP (2001–2004).

	MEHP		MEHHP		MEOHP		Molar sum of MEHP, MEHHP, and MEOHP	
	Cases/*n*	OR 95% CI	Cases/*n*	OR 95% CI	Cases/*n*	OR 95% CI	Cases/*n*	OR 95% CI
Endometriosis (*n*= 838)								
Lowest three quartiles^b^	45/615	1.00 Reference	47/628	1.00 Reference	46/627	1.00 Reference	47/629	1.00 Reference
Highest quartile^b^	12/223	0.60 (0.24–1.50)	10/210	0.46 (0.18–1.21)	11/211	0.62 (0.27–1.44)	10/209	0.48 (0.18–1.25)
Uterine leiomyomata (*n*= 835)								
Lowest three quartiles^b^	91/612	1.00 Reference	87/626	1.00 Reference	84/625	1.00 Reference	86/627	1.00 Reference
Highest quartile^b^	22/223	0.66 (0.32–1.37)	26/209	0.97 (0.55–1.71)	29/210	1.43 (0.87–2.37)	27/208	1.08 (0.60–1.95)
Endometriosis or uterine leiomyomata (*n*= 835)								
Lowest three quartiles^b^	115/612	1.00 Reference	116/626	1.00 Reference	113/625	1.00 Reference	115/627	1.00 Reference
Highest quartile^b^	31/223	0.69 (0.38–1.25)	30/209	0.69 (0.43–1.12)	33/210	0.96 (0.63–1.47)	31/208	0.77 (0.46–1.29)

a Adjusted for age, race/ethnicity, age at menarche, and current pregnancy status. Further adjustment for current breast-feeding status created model instability.

b Ranges for MEHP groups (ng/mg): 0.2–6.3 (lowest three quartiles) and 6.4–538 (highest quartile); ranges for MEHHP groups (ng/mg): 0.5–31.9 (lowest three quartiles) and 32.0–1885 (highest quartile); ranges for MEOHP groups (ng/mg): 0.6–21.3 (lowest three quartiles) and 21.4–1115 (highest quartile); and ranges for molar sum of MEHP, MEHHP, and MEOHP (nmol/mg): 0.6–27.5 (lowest three quartiles) and 27.5–1094 (highest quartile).
